# Innovative cardiovascular casting technique features the complex malformation of berry syndrome

**DOI:** 10.1186/s12884-024-06340-2

**Published:** 2024-03-12

**Authors:** Wei Li, Wei Feng, Caihong Chang, Ya Liu, Xue Li, Mofeng Wang, Ling Gan, Jiaqi Zhang

**Affiliations:** 1 Hubei Provinical Clinical Research Center for Accurate Fetus Malformation Diagnosis, Department of gynaecology and obstetrics, Xiangyang No. 1 People’s Hospital, Hubei University of Medicine, No 15, Jiefang Avenue, Xiangyang, 441000 China; 2 Depatment of Ultrasound, Hubei University of Medicine, Xiangyang No. 1 People’s Hospital, Xiangyang, 441000 China; 3 Department of Ultrasound, Hubei University of Medicine, Xiangyang No.1 People’s Hospital, No 15, Jiefang Avenue, Xiangyang, 441000 Hubei China

**Keywords:** Berry syndrome, Complex cardiac malformation, Prenatal diagnosis, Postpartum

## Abstract

**Background:**

Prenatal diagnosis of Berry syndrome, a rare combination of cardiac anomalies including aortopulmonary window (APW), aortic origin of the right pulmonary artery (RPA), interrupted aortic arch (IAA), hypoplastic aortic arch, or coarctation of the aorta (COA), poses a significant challenge. Due to the rarity of the disease, and the limited case reports available to features the complex malformation of Berry syndrome postpartum, this article introduces an innovative approach to visually showcase this unusual disease. The proposed method provides a comprehensive display of the structural deformities, offering valuable insights for clinical practitioners seeking to comprehend this condition.

**Case presentation:**

In this report, we present a case where fetal echocardiography aided in diagnosing Berry syndrome, which was later confirmed through postpartum cardiovascular casting. Our experience highlights the importance of using the three-vessel view to diagnose APW and aortic origin of the right pulmonary artery. Additionally, obtaining true cross-sectional and sagittal views by continuously scanning from the three-vessel-trachea view to the long-axis view of the aortic arch is necessary to image IAA or coarctation of the aortic arch.

**Conclusions:**

Early and accurate prenatal diagnosis of Berry syndrome is feasible and our cardiovascular cast can perfectly display the microvascular morphology of the fetal heart, which may have great application prospects for postpartum diagnosis and teaching of complex cardiac abnormalities.

**Supplementary Information:**

The online version contains supplementary material available at 10.1186/s12884-024-06340-2.

## Introduction

Berry syndrome is an extremely uncommon congenital cardiac malformation characterized by several abnormalities, including an aortopulmonary window (APW), aortic origin of the right pulmonary artery (RPA), and interruption or coarctation of the aortic arch (IAA), with an intact ventricular septum. The first report of Berry syndrome was by Berry et al. in 1982 [1], and only a few cases have been diagnosed prenatally since then, with the first prenatal echocardiographic diagnosis being reported by Matsubara et al. in 2010 [2–4]. Due to its rarity and complexity, prenatal diagnosis of Berry syndrome is particularly challenging [5, 6]. Our center is a maternal-fetal medicine consultation center for fetal cardiac disease. Many gravidas have been transferred to our center for diagnosis and counseling when their fetuses were suspected to have cardiac anomaly, so we have experience in diagnosing rare fetal heart abnormalities. Here, we report a case in which Berry syndrome is diagnosed by fetal echocardiography and confirmed by postpartum cardiovascular casting. In this case report, we provide an in-depth analysis of the distinctive features of Berry syndrome and highlight the critical aspects of prenatal ultrasound diagnosis. Additionally, we employed advanced cardiovascular technology to construct a cardiovascular cast model, which enhances our understanding of this complex cardiac malformation [7–10].

Our findings suggest that fetal echocardiography is the preferred diagnostic tool for Berry syndrome, and accurate diagnosis can be achieved by utilizing the three-vessel view and cross-sectional and sagittal views. Furthermore, we applied a novel perfusion technique to create a fetal cardiovascular model, which provides valuable postpartum validation and teaching opportunities. This technology enables us to visualize the shape and spatial relationship of the cardiovascular system in a comprehensive and accurate manner, enhancing our understanding of complex fetal heart diseases.

## Case report

**Fig. 1:**
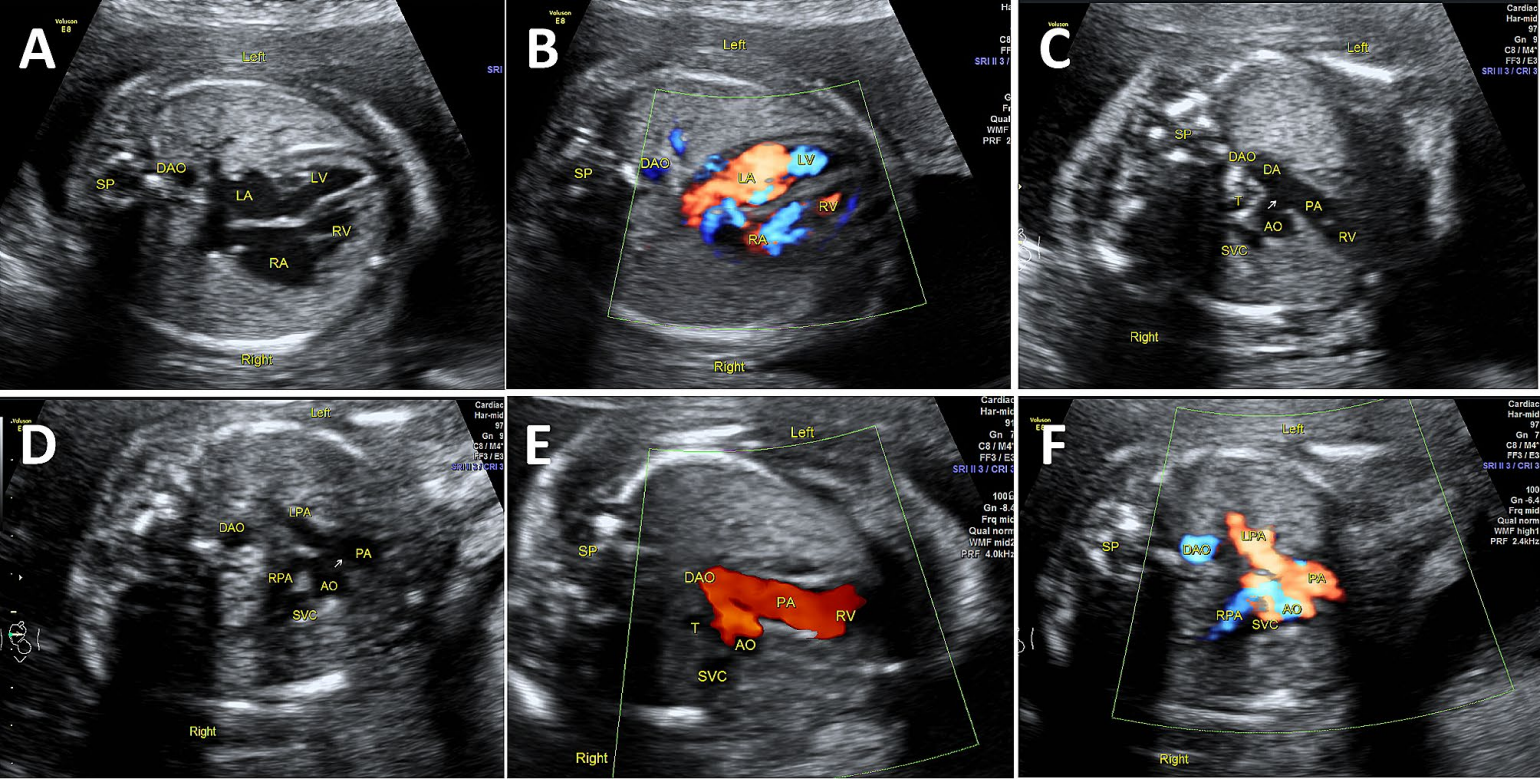
**(A, B)** Four-chamber view shows normal position of the heart and cardiac axis. The size of the left and right atria and ventricles are also normal, and the interventricular septum is intact. No shunting blood flow is observed across the interventricular septum. DAO, descending aorta. **(C-F**) On a three-vessel view, the ascending aorta is connected to the MPA, forming the APW. The anomalous origin of the RPA originates from the AAO, while the LPA originates from the autonomous PA. AAO, ascending aorta; LA, left atrium; LV, left ventricle; LPA, left pulmonary artery; PA, pulmonary artery; RA, right atrium; RV, right ventricle; SP, spleen; SVC, superior vena cava; T, trachea

**Fig. 2:**
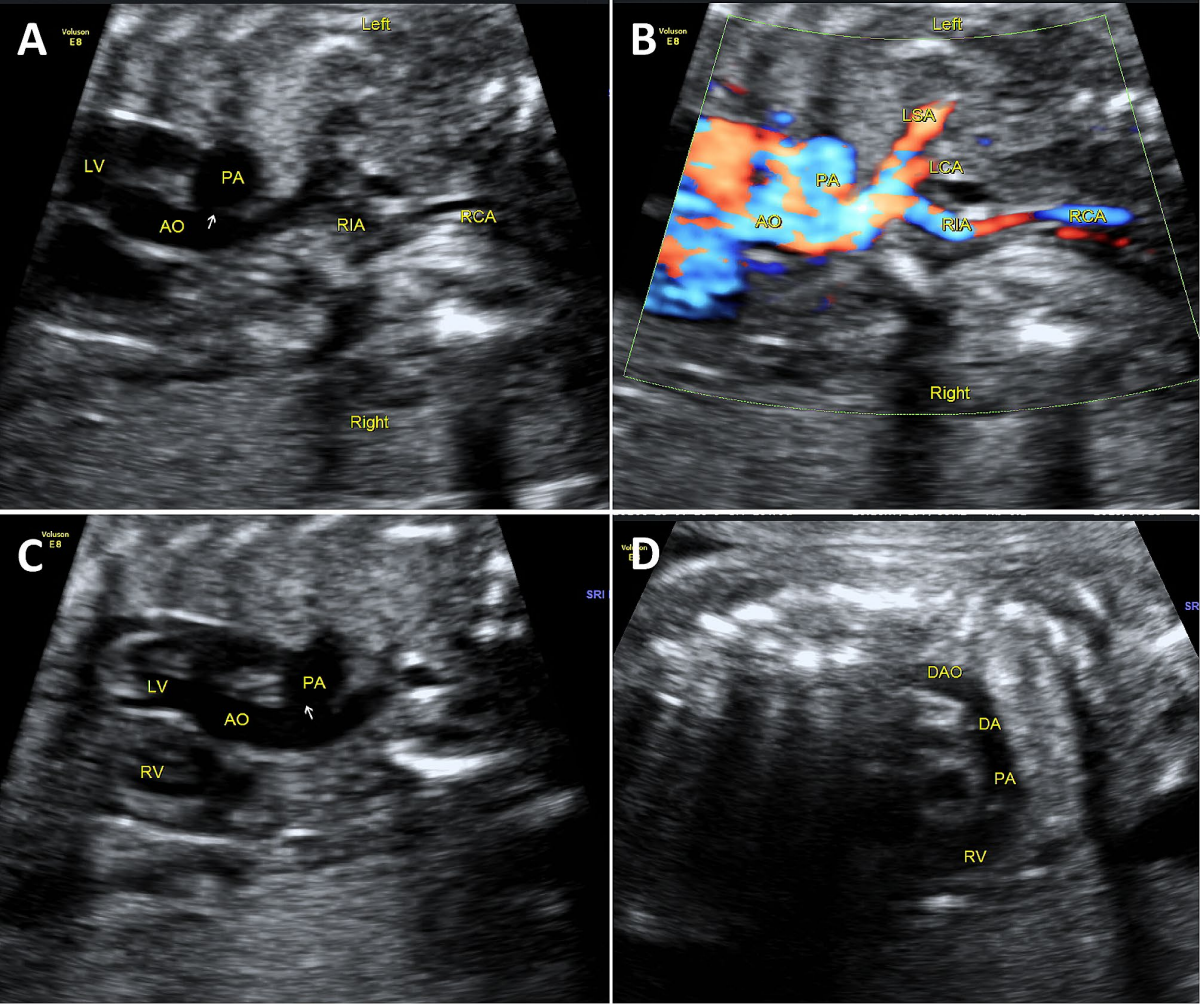
**(A, B)** The AAO appears thin in the long axial section of the aortic arch, and following it, the RIA, LCA, and LSA branch off successively. A visible interrupt can be seen behind the LSA. The APW is indicated by an arrow. **(C)** In the left ventricular outflow tract section, the AAO originates from the LV and is connected to the PA. **(D)** The distal end of the AO is thinner. In the long axis section of the AO, the PA originates from the right ventricle and continues backwards as an DA. APW, aortopulmonary window; DA, ductus arteriosus; LCA, left common carotid artery; LSA, left subclavian artery; RCA, right common carotid artery; RIA, right brachiocephalic trunk artery

A 30-year-old pregnant woman at 23 weeks of gestation was referred to our hospital for suspected fetal cardiac malformations. The prenatal diagnosis at 23 weeks revealed complex congenital heart disease in the fetus, which included an observation of the cardiac crux and intact ventricular septum (Fig. 1A, B). Additionally, the ascending aorta was found to be disconnected from the descending aorta, indicating interruption aortic arch, type A. The fetal echocardiography of the three-vessel trachea view (3VT) showed that the ascending aorta was connected with the main pulmonary artery, and the right pulmonary artery originated from the aorta (Fig. 1C-F **and Supplementary Videos** S1). Furthermore, the ascending aorta appears thin in the long axial section of the aortic arch, and following it, the cephalbrachial trunk, left common carotid artery, and left subclavian artery branch off successively (Fig. 2A, B). The interruption of aortic arch and the blood flow interruption were confirmed by the long-axial view of the aortic arch and left ventricular outflow tract (Fig. 2C, D **and Supplementary Videos** S2). The pregnancy was terminated at the gestational age of 28 + 3 weeks. The family voluntarily donated the specimen to our maternal and fetal medical center and provided written informed consent for this study.

The fetal specimen has been donated to our Maternal Fetal Medicine Center and underwent a detailed step-by-step process to create a cardiovascular model.

1. Filling agents.

Self-setting dental tray liquid and powder, Acrylonitrile-Butadiene-Styrene Copolymer (ABS), Heparin, red and blue dye.

2. Catheterization equipment.

In the production of fetal molded specimens, the choice of catheterization is crucial. Given the delicate nature of fetal vessels and organ channels, which can be easily punctured by regular needles, we utilized soft catheter materials modified from infusion tubing and needles to avoid piercing the fragile fetal vessels.

3. Catheter selection and techniques for infusing different fetal organs:

Selecting fresh fetal specimens aged 20 weeks or more, the fetus is positioned according to anatomical posture. Due to the involvement of various systems and organs in fetal casting, and the different compositions of filling agents used in perfusion, the following steps are taken: [1] Open the chest and abdominal cavities along the midline from the fetal mandible to the upper margin of the pubic symphysis [2]. Dissect the umbilical vein and one side of the umbilical artery at the navel, inserting self-made plastic catheters and ligating them [3]. Gently peel off the thymus above the heart, ligating the ductus arteriosus and aortic arch near the heart. Clamp the trachea below the cartilage with hemostatic forceps, insert a bent injection needle along the trachea, and block the passage between the respiratory and digestive tracts [4]. Insert a plastic catheter through the oral cavity into the stomach, clearing meconium through a colon incision due to the presence of fetal feces.

4. Sequence and Volume of Infusion:

Firstly, open the abdominal wall, dissect the umbilical vein, and insert a small tube. Then, separate and incise the umbilical artery on the left side. Inject heparin (5–10 mL) and acetone (20–50 mL) successively through the umbilical vein to clear thrombi from the cardiovascular system. Next, inject the sample mixture, including Acrylonitrile-Butadiene-Styrene Copolymer (ABS) (30–60 mL), dental tray powder (dissolved in purified water at a 1:1 ratio), and dental tray liquid (at a ratio of 3:1:1). This injection process needs to proceed at appropriate pressure and a constant rate for about 10 h. Additionally, red and blue dye (0.5 mL each) is added for enhanced visualization. After 24 h, the perfused specimen is immersed in 30% hydrochloric acid. Two weeks later, carefully remove the sample from the hydrochloric acid. Finally, label, display, and evaluate the fetal cardiovascular model to obtain accurate and detailed anatomical information.

5. Suggestions for Specimen Rinsing Stage:

Based on our perfusion experience, to avoid damage to microstructures such as micro-vessels, it is necessary to continuously adjust the water flow during the rinsing process. Initially, the rinsing volume should be relatively large, gradually decreasing over time, with adjustments made approximately every 3 min. The rinsing sequence involves initially flushing the fetal atria and ventricles, followed by the arteries and veins.

**Fig. 3:**
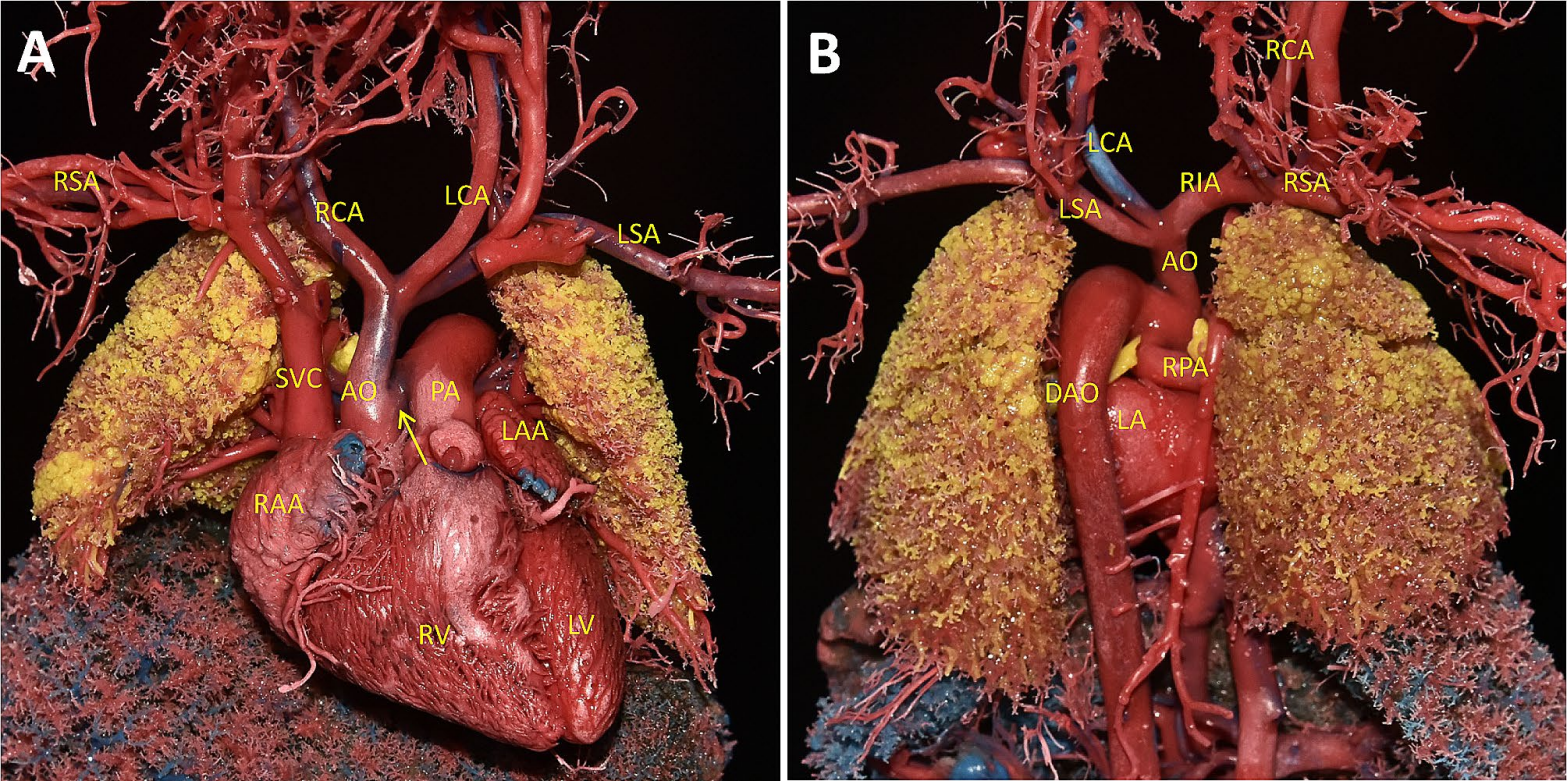
Cardiovascular cast of Berry syndrome. **(A)** From the front view of the casting, the aortic arch was interrupted, IAA type A and connects the AAO with the main PA. **(B)** Seen from the back, the aortic arch was interrupted and connects the AAO with the MPA. The RPA originated from the AO.

**Fig. 4:**
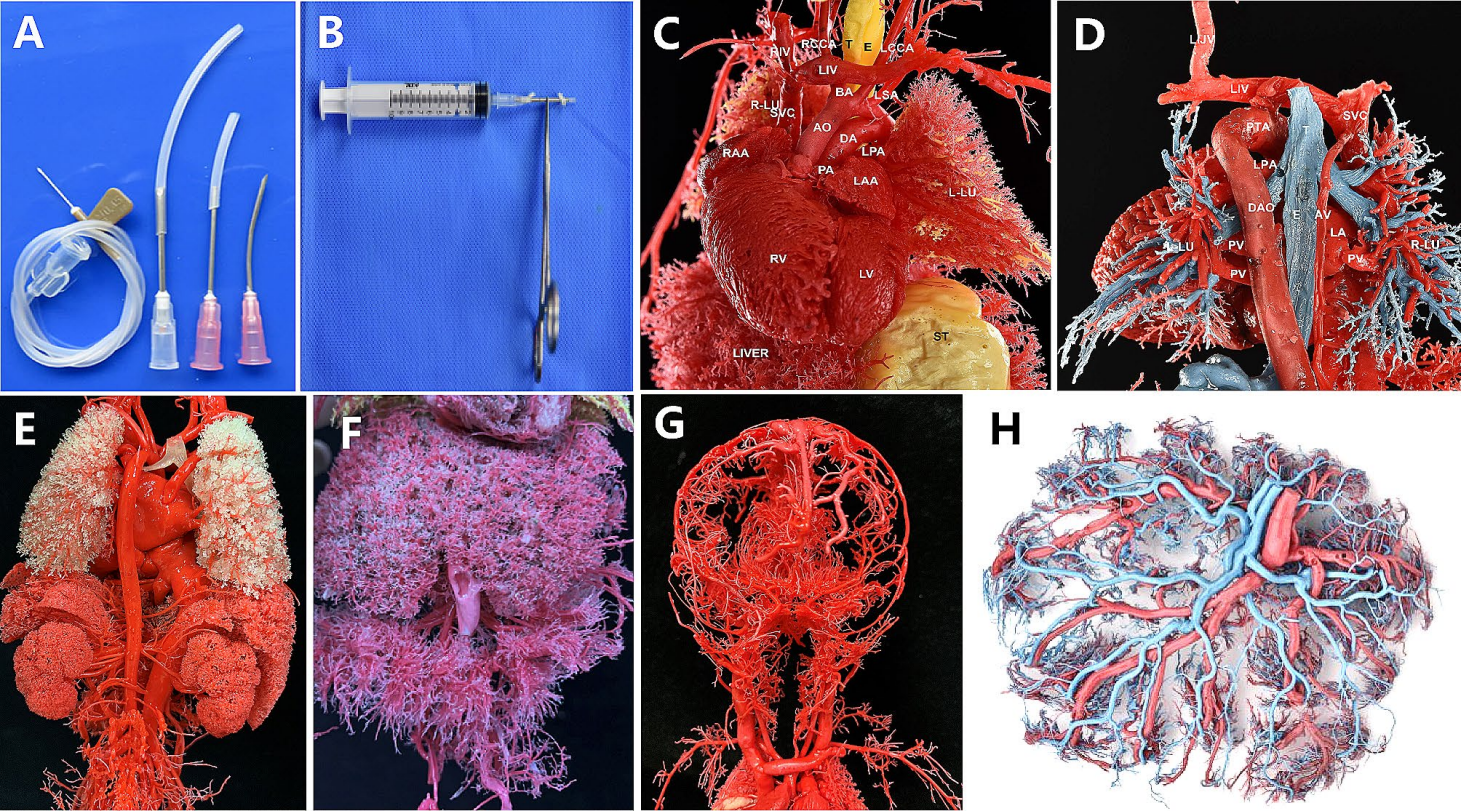
Our innovative cardiovascular casting technique is capable of revealing the structure and spatial distribution of micro-vessels in different organs associated with the fetus. **(A,B)** Homemade vascular perfusion tubes and instruments;(**C)** Cardiovascular casting model; **(D)** Pulmonary vascular cast model; **(E)** Kidney and adrenal cast model; **(F)** Liver and mesenteric cast model; **(G)** Fetal cranial cast model; **(H)** Placental casting model

Subsequent anatomy and a cardiovascular cast confirmed the prenatal diagnosis **(**Fig. 3A, B**)**. Our casting technology is capable of revealing the structure and spatial distribution of micro-vessels in different organs associated with the fetus (Fig. 4A-H).

## Discussion

Berry syndrome is a rare subtype of IAA that is associated with APW and occurs in approximately 0.046% of the population with congenital cardiac malformations [11]. Early diagnosis and timely surgical intervention are necessary to restore normal perfusion, particularly to the lower body, and to prevent pulmonary damage [12]. Infants, who make up the majority of the approximately 40 reported cases in the literature, typically present with low cardiac output syndrome and respiratory distress, similar to those with ductal-dependent systemic circulation resulting from severe left-sided obstructive cardiac lesions [1, 11–13]. Successful surgical repair of this complex congenital cardiac anomaly has been reported in both infants and older children, using a single- or multi-staged approach [12–17].

**Fig. 5:**
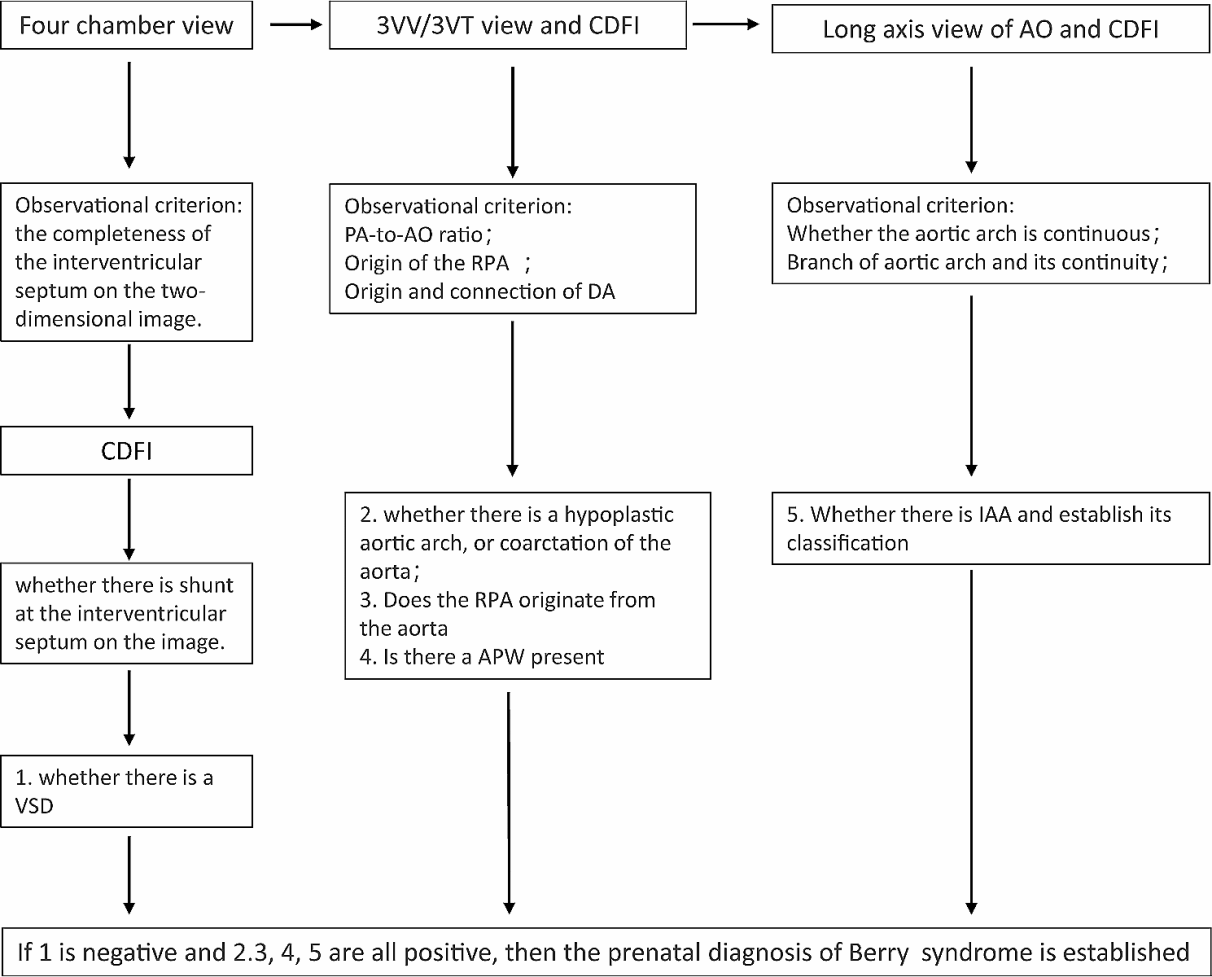
The prenatal ultrasound diagnostic approach for Berry syndrome

Prenatal diagnosis of Berry syndrome is challenging due to the multiple complex deformities involved, which require performing multiple cross-sectional scans during prenatal ultrasound to achieve a comprehensive diagnosis. To date, only eight cases of prenatally diagnosed Berry syndrome have been reported in the literature. Furthermore, there is a lack of postpartum validation images in most cases as is shown in Table 1 [2, 3, 5, 6]. The difficulty in diagnosis of Berry syndrome is primarily due to the normal four-chamber view and ventricular outflow tract views, without associated intracardiac septal defects. Additionally, the aortic origin of RPA can be easily missed, especially if pulmonary artery branching is not carefully assessed. The ductus arteriosus arch may also be mistaken as the aortic arch in the long-axis view, leading to a missed diagnosis of IAA or COA. In the normal fetal heart, the 3VT view displays from left to right: the PA, aortic arch, trachea, and SVC. The PA converges with the aortic arch through the DA into the descending aorta, without forming an APW **(Supplementary Fig. **1A). In the long-axis section of the aortic arch, the arch appears “cane-shaped,” with three branches sequentially identified as the RIA, LCA, and LSA. The descending aorta, ascending aorta, and aortic arch continue seamlessly without sudden narrowing or interruption **(Supplementary Fig. **1B). Cardiovascular casting provides a more intuitive representation of this normal anatomical structure **(Supplementary Fig. **1C, D).The three-vessel view assessment is critical in prenatal diagnosis, as it reveals the defect between the aorta and the pulmonary trunk, the origin of RPA from the AAO, the origin of LPA from the pulmonary trunk, and an abnormal PA-to-AO ratio [6]. Therefore, it is essential to continuously scan from the 3VT to the long-axis view of the aortic arch, with the help of color and pulsed Doppler echocardiography to demonstrate the true cross-sectional and sagittal views. Another important aspect of prenatal diagnosis is the detection of an APW, which is often the first abnormal sign in fetuses with Berry syndrome. When an APW is detected, it is imperative to define the origin of the RPA and the morphology of the aortic arch to confirm the diagnosis of Berry syndrome **(**Fig. 5**)**. In clinical practice, Berry syndrome should primarily be differentiated from APW and interrupted aortic arch. Berry syndrome represents one of the pathological subtypes of APW, with a key distinguishing factor being the presence of inadequate development or interruption of the aortic arch in Berry syndrome cases. In the case of interrupted aortic arch, the main differentiating point lies in the fact that in interrupted aortic arch cases, the aorta and pulmonary artery are independent of each other and do not form an APW **(Supplementary Fig. **2).

**Table 1:** Summary of 8 cases of Berry syndrome diagnosed prenatally

Author	Maternal age	Gestational age	Prenatal diagnosis	Postpartum outcome	Postpartum image
Xin Zhang et al.	32	24	Berry syndrome, the RPA originate from the posterior wall of the AAO, with normal origin of the left pulmonary artery (LPA), and a 7.4 mm defect was found between the aorta AO and MPA.A bidirectional shunt between the AAO and MPA. The transverse aortic arch was hypoplastic, coarctation of the aorta (COA)	Termination in late pregnancy and made fetal autopsy	No
Xin Zhang et al.	27	27	Berry syndrome, the 3VV and 3VT views revealed a 4.5 mm defect between the AO and the MPA.	Terminated and fetal autopsy	No
Xin Zhang et al.	31	29	Berry syndrome with 3VV and 3VT of fetal echocardiogram showed a 5.0 mm APW	Terminated the pregnancy but did not agree to an autopsy	No
Xin Zhang et al.	28	23	Berry syndrome, the fetal echocardiogram revealed a 3.5 mm APW, the origin of RPA from the posterior wall of the AAO, and type A IAA.	Lost to follow-up	No
Yang et al.	26	24	Berry syndrome, AAO was not connected to DAO and SVC was found on the left of PA. The presence of interrupted arch and intact ventricular septum was concerning for coexisting APW. An IAA (type A) was confirmed.	requested a termination of late pregnancy and agreed to an autopsy.	Yes
Sunil.Ghelani et al.	26	25	A fetal echocardiogram at 25 weeks’ gestation demonstrated the constellation of Berry syndrome	Surgical repair was performed at 13 days of age.	Yes
O Poujada et al.	30	24 + 4	Berry syndrome, a perfusion from the AOO to the PA and IAA. The DA was directly connected to the DAO. The origins of the LPA were spread apart, assuming a shape similar to the barbs of a catfish on fetal echocardiography	Surgical repair was performed on the second day and thirteenth day after deliery respectively .	No
Jiang Wu et al.	28	26	Berry syndrome, A 6-mm defect between the AO and pulmonary trunk in the left outflow tract and aortic arch was detected. Bidirectional shunt flow, indicating an APW). Normal locations of the AO and PA; the RPA originated from the AAO, and the LPA originated from the pulmonary trunk; A gradual decrease in the aortic diameter and IAA after the origin of its 3 branches. The DAO was connected to the DA, which originated from the PA trunk; An intact ventricular septum and normal mitral and tricuspid valves were shown	The neonate developed pneumonia and heart failure and died 63 days postnatally after his family stopped all treatments.	No

PW: aortopulmonary window; Ao: aorta; MPA: main pulmonary artery; IAA: interrupted aortic arch, LPA: left pul-monary artery, RPA: right pulmonary artery; PDA: patent ductus arteriosus; DAO: descending aorta.2 H. BU AND T. ZHAO

In summary, although prenatal diagnosis of Berry syndrome is challenging, it is essential to detect this rare congenital cardiac anomaly early to improve outcomes. The 3VV assessment, color- and pulsed Doppler echocardiography, and detection of an APW are crucial in the diagnosis of Berry syndrome prenatally.

## Conclusion

In our experience, the 3VV and 3VT views have been found to be the most important and useful views for antenatal diagnosis of Berry syndrome. In cases where an APW is present, it is essential to clearly define the origins of the pulmonary arteries and the morphology of the aortic arch in order to improve the accuracy of diagnosis.

Furthermore, our improved cardiovascular cast has the ability to clearly depict the morphology of large vessels and small branches, providing a detailed three-dimensional structure of the heart. This method shows promise as a supplementary approach for the postmortem assessment of fetal congenital heart malformations in the future.

### Electronic supplementary material

Below is the link to the electronic supplementary material.

Supplementary Material 1

Supplementary Material 2

Supplementary Material 3

Supplementary Material 4

Supplementary Material 5

## Data Availability

The original contributions presented in the study are included in the article/ Supplementary materials. Further inquiries can be directed to the corresponding author.

## Abbreviations

AAO: Ascending aorta
ABS: Butadiene-styrene plastic
APW: Aortopulmonary window
COA: Coarctation of the aorta
DA: Ductus arteriosus
IAA: Interrupted aortic arch
LA: Left atrium
LCA/LCCA: Left common carotid artery
LPA: Left pulmonary artery
LSA: Left subclavian artery
LV: Left ventricle
PA: Pulmonary artery
RA: Right atrium
RCA/RCCA: Right common carotid artery
RIA: Right brachiocephalic trunk artery
RPA: Right pulmonary artery
RV: Right ventricle
SP: Spleen
SVC: Superior vena cava
T: Trachea
3VV: Three vessel view
3VT: Three-vessel trachea view
