# Supplementary figures and images for: Innovative cardiovascular casting technique features the complex malformation of berry syndrome
Source: BMC Pregnancy Childbirth. 2024 Mar 12;24:194. doi: 10.1186/s12884-024-06340-2 (PMC10935913; doi:10.1186/s12884-024-06340-2)

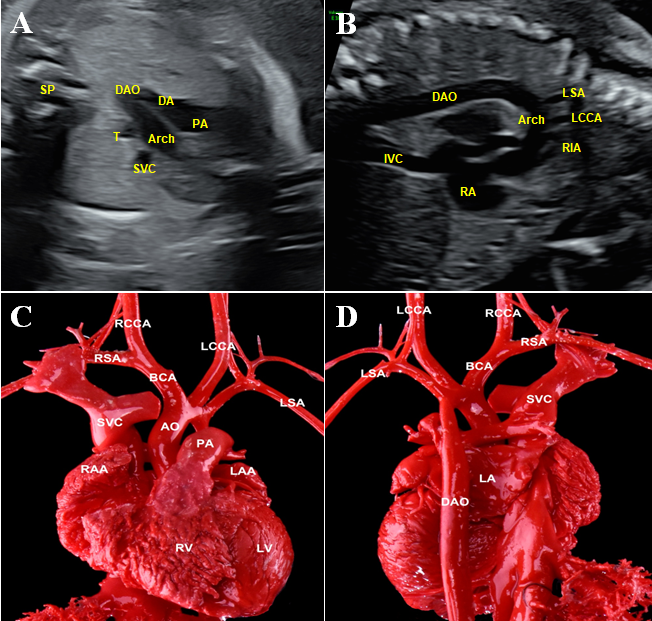

Supplement: Supplementary file 3 — Supplementary Material 3 [file 12884_2024_6340_MOESM3_ESM.png]

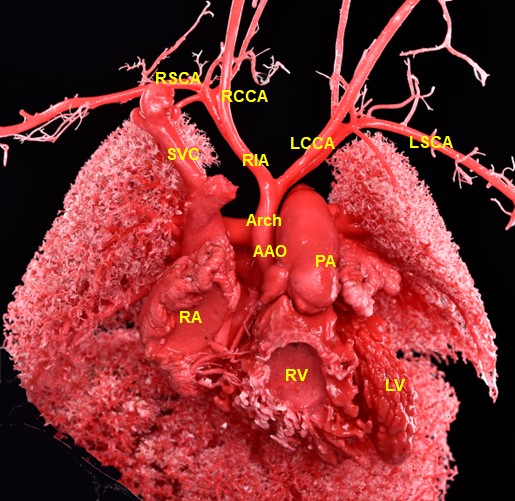

Supplement: Supplementary file 4 — Supplementary Material 4 [file 12884_2024_6340_MOESM4_ESM.png]
