# Supplementary material for: Innovative cardiovascular casting technique features the complex malformation of berry syndrome
Source: BMC Pregnancy Childbirth. 2024 Mar 12;24:194. doi: 10.1186/s12884-024-06340-2 (PMC10935913; doi:10.1186/s12884-024-06340-2)
Supplement: Supplementary file 5 — Supplementary Material 5 [file 12884_2024_6340_MOESM5_ESM.docx]

**Legend of supplementary materials**

**Figure 1:** 3VT view **(A)**, long-axis view of the aortic arch **(B)** and cardiovascular casting **(C, D)** of the normal fetal heart.

**Figure 2:** Cardiovascular casting manifestation of IAA (Type A).

**Videos: S1-S2.**
